# STAT3 gain-of-function syndrome

**DOI:** 10.3389/fped.2022.770077

**Published:** 2023-02-09

**Authors:** Tiphanie P. Vogel, Jennifer W. Leiding, Megan A. Cooper, Lisa R. Forbes Satter

**Affiliations:** ^1^ Department of Pediatrics, Baylor College of Medicine and William T. Shearer Center for Human Immunobiology, Texas Children's Hospital, Houston, TX, United States; ^2^ Division of Allergy and Immunology, Department of Pediatrics, Johns Hopkins University, Baltimore, MD, United States; ^3^ Orlando Health Arnold Palmer Hospital for Children, Orlando, FL, United States; ^4^ Division of Rheumatology and Immunology, Department of Pediatrics, Washington University School of Medicine, St. Louis, MO, United States

**Keywords:** STAT3, lymphoproliferation, early onset autoimmunity, immune dysregulation, autoimmune cytopenia

## Abstract

STAT3 gain-of-function (GOF) syndrome is a multi-organ primary immune regulatory disorder characterized by early onset autoimmunity. Patients present early in life, most commonly with lymphoproliferation, autoimmune cytopenias, and growth delay. However, disease is often progressive and can encompass a wide range of clinical manifestations such as: enteropathy, skin disease, pulmonary disease, endocrinopathy, arthritis, autoimmune hepatitis, and rarely neurologic disease, vasculopathy, and malignancy. Treatment of the autoimmune and immune dysregulatory features of STAT3-GOF patients relies heavily on immunosuppression and is often challenging and fraught with complications including severe infections. Defects in the T cell compartment leading to effector T cell accumulation and decreased T regulatory cells may contribute to autoimmunity. While T cell exhaustion and apoptosis defects likely contribute to the lymphoproliferative phenotype, no conclusive correlations are yet established. Here we review the known mechanistic and clinical characteristics of this heterogenous PIRD.

## Introduction

Investigation of patients with inborn errors of immunity (IEI) has provided insight into the human immune system and mechanisms governing regulation of a coordinated and controlled response (1, 2). Such defects of human immunity can present with a broad range of clinical symptoms, including infectious susceptibility, autoinflammation, autoimmunity, and cancer pre-disposition. Gain-of-function (GOF) variants in *STAT3*, encoding signal transducer and activator of transcription 3 (STAT3), lead to a syndrome of early-onset autoimmunity with immune dysregulation first recognized in 2014 (3). Since the initial discovery of the molecular mechanism of STAT3 GOF syndrome, further identification and investigation of these patients has increased understanding of the often severe clinical consequences of this disease and the effects of abnormal STAT3 regulation on immunity, and provided rationale for targeted therapeutic approaches (4). Here we review our current knowledge of the mechanisms and clinical course of this immune dysregulation syndrome.

### STAT3 biology

**Figure 1:**
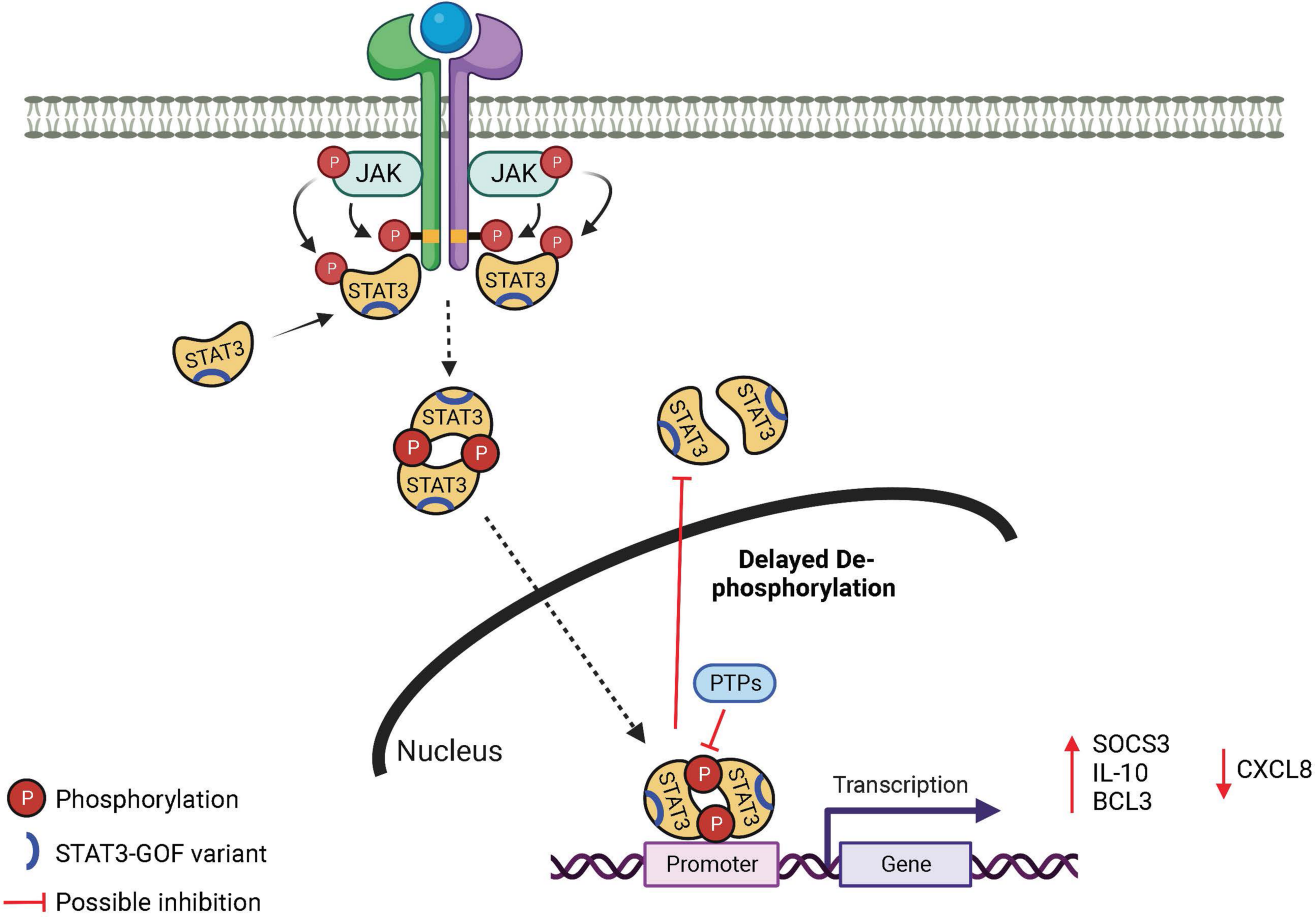
Mechanism in STAT3 GOF syndrome. JAK, Janus kinase; STAT, signal transducer and activator of transcription; PTP, protein tyrosine phosphatase.

STAT3 belongs to a family of 7 STAT proteins and is found in immune and non-immune cells. It is a critical regulator of cellular survival, proliferation, differentiation, and effector function. Cytokines including the IL-6 and IL-10 families and IL-21, IL-23, and IL-27 activate STAT3 (Figure 1). After cytokines bind to their respective receptors, Janus kinases (JAKs) are activated, which in turn leads to STAT3 phosphorylation. Phosphorylated STAT3 translocates into the nucleus where it binds to specific DNA sequences.

### Mechanism of STAT3 GOF variants confer increased activity

Determining the underlying mechanisms behind how single amino acid changes (and one single deletion) in the STAT3 protein functionally lead to a GOF is an area of active investigation given the insights such an understanding would provide. The generally accepted standard to determine if a genetic variant in a transcription factor, like STAT3, has altered function is to use a transcriptional assay, most commonly a reporter system such as luciferase (5). For STAT3, this entails co-transfecting a plasmid that will lead to expression of the STAT3 variant of interest with a reporter construct containing STAT3 binding sites upstream of a luciferase gene in a cell line lacking endogenous STAT3 activity. Subsequent luciferase production is a measurable read-out, and generated in proportion to the transcriptional capacity of the STAT3 variant of interest. In this manner, nearly every reported STAT3 GOF variant has been confirmed to lead to a gain-of-function at baseline or with cytokine stimulation (5, 6). It remains to be seen what further factors may explain the wide range in transcriptional power, anywhere from 2 to 100 times more than wild-type, of various STAT3 GOF variants (5, 6).

A luciferase assay allows for distinction of GOF variants from STAT3 polymorphisms that do not change transcriptional function, or variants that lead to loss-of-function (3, 5). However, a transfection system may not sufficiently reflect the biology of a complex and protean molecule like STAT3 in immune or other cells *in vivo*. For instance, despite the fact that most STAT3 GOF variants have much greater than wild-type transcriptional capacity in a luciferase assay system at baseline, STAT3 GOF variants do not appear to lead to constitutive activation (*i.e.*, phosphorylation) of STAT3 in peripheral blood mononuclear cells (PBMCs) of patients with STAT3 GOF syndrome, *e.g.,* in cells that are not transfected or transformed (5, 7–11). Even with stimulation, PBMCs from STAT3 GOF patients rarely demonstrate hyper-phosphorylation (12), unlike in STAT1 GOF where hyper-phosphorylation in primary cells can identify disease-causing variants (13). Indeed, STAT3 GOF variants can be under-phosphorylated compared to wild-type (5, 7).

STAT3 GOF variants are found throughout the molecule, encompassing each of the protein domains (14). There is growing evidence in both primary and patient-derived cells that activation of a number of DNA-binding domain (DBD) STAT3 GOF variants leads to a prolonged activation state, specifically delayed de-phosphorylation over time (5, 6) (Figure 1). This may be due to augmented DNA binding and nuclear retention (6) as the result of the specific amino acid changes, which have been predicted to result in enhanced DNA interactions due to replacement with more positively charged residues (3, 15). It is not clear this mechanism will be true for STAT3 GOF variants in other domains, as N-terminal variants do not appear to bind DNA stronger (6), and coiled-coil domain (CCD) variants do not appear to have altered nuclear kinetics (6, 16).

Measuring transcriptional capacity using luciferase provides only one terminal read-out, and may not entirely account for non-canonical roles of STAT3. Non-canonical mechanisms (17) could explain why some rare and predicted damaging STAT3 variants in patients with convincing clinical phenotypes do not generate significantly higher than wild-type luciferase results (5, 6). Non-canonical signaling or non-transcriptional roles for STAT3 GOF variants have not been investigated thus far.

Upregulation of several known STAT3 targets has been investigated. Most frequently reported is *SOCS3*, which is increased at baseline and after stimulation in most reports with similar experimental design (5, 6, 18, 19). It has been postulated that the increase in *SOCS3* results in negative feed-back on the activation of other STAT molecules (5, 8, 15, 20), including STAT5 which plays a role downstream of growth hormone. Other target genes have also been noted to differ from wild-type after stimulation of STAT3 GOF molecules including enhanced (IL-10, BCL3) (6, 21) and decreased (CXCL8) (16) expression (Figure 1). It may be that increased expression of pro-survival genes downstream of STAT3 GOF variants (18) contributes to an apoptosis defect that drives lymphoproliferation, one of the most common clinical findings.

How an enhanced STAT3 GOF transcriptional signature is connected to the clinical and immunological phenotypes of STAT3 GOF patients is starting to grow clearer. Much attention has been given to the CD4+ T cell populations in STAT3 GOF patients due to the known role of IL-6 signaling in T regulatory and Th17 cell development and the phenotypic connection of the patients with others that carry T regulatory cell defects (14). Enhanced STAT3 signaling would be predicted to result in elevated CD4+ Th17 cells (22), however, while it is clear that patients with STAT3-hyper-IgE-syndrome due to dominant negative STAT3 loss-of-function variants have decreased CD4+ Th17 cells (23), it remains unclear if the corollary of enhanced Th17 cells is present in STAT3 GOF patients (4). This may be secondary to the treatment status of patients at the time of immune profiling, as immune modulation can impact the differentiation of CD4+ T cells (24). When enumerated, CD4+ T regulatory cells have been noted to be decreased in STAT3 GOF patients (4, 5, 9, 16, 20, 25, 26), which may also have decreased function (10). However, recently, a murine model for STAT3 GOF generated through CRISPR/Cas9 genetic engineering in a diabetic prone mouse model showed that disease can be driven by a cell intrinsic defect in CD8+ T cells which do not progress through to terminal exhaustion and then drive pathology (27). Another murine model of STAT3 GOF, reported oligoclonal accumulation of effector CD8+ T cells that contributed to autoimmunity (28). Two additional murine models demonstrated no functional differences in T regulatory cells, but suggested enhanced Th1 polarization (29, 30) and IFN-*γ* production by CD4+ T cells. The lack of a profound defect of T regulatory cells was supported by single cell transcriptional analysis of cells from patients (30). Interestingly, a patient with PD-1 deficiency was recently described to have susceptibility to *Mycobacterium tuberculosis* with an immunologic phenotype similar to STAT3 GOF, including expanded DNTs thought to be the result of increased STAT3-mediated signaling, suggesting a possible explanation for mycobacterial disease in some STAT3 GOF patients (31).

Finally, while it has been reported in a collection of patients (5, 6), no explanation yet exists for the incomplete penetrance of STAT3 GOF variants in certain families with asymptomatic carriers. This suggests that there are other disease modifiers, including genetic and environmental, that will be important to identify in order to understand the mechanisms of immune dysregulation in these patients.

### Clinical manifestations of STAT3 GOF

**Figure 2:**
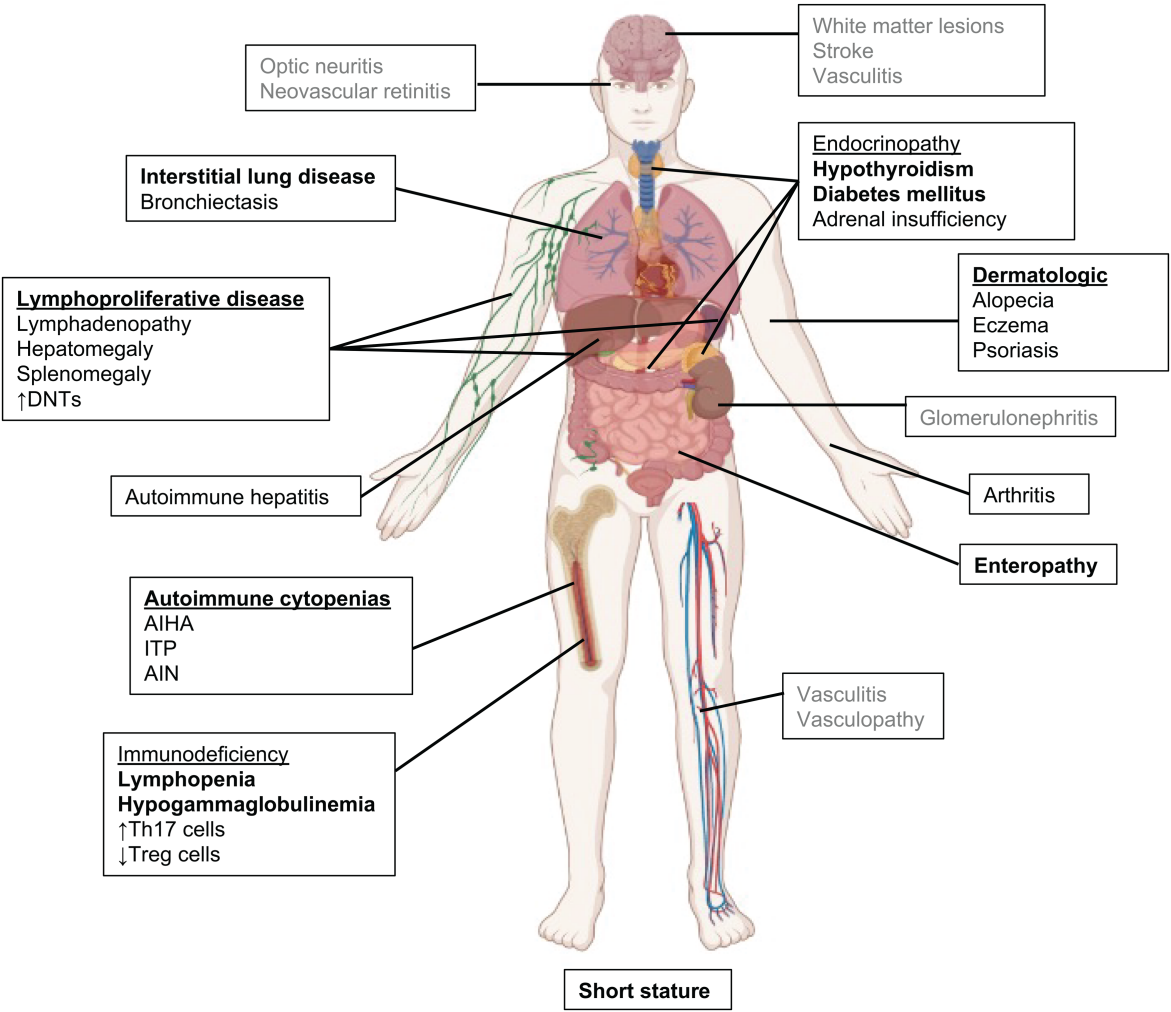
Clinical manifestations in STAT3 GOF syndrome. The most common features are in bold, rarely reported features are in gray.

The original clinical descriptions of patients with STAT3 GOF included early onset type I diabetes mellitus, post-natal growth failure, and lymphoproliferation (3, 5, 10). Although all 3 remain hallmark symptoms of the disease, the clinical and immune phenotype has significantly expanded as additional patients have been identified (4) (Figure 2). Onset of disease is early, occurring at a median of 2.3 years (4, 14). Early onset diabetes, autoimmune cytopenias, enteropathy, and growth failure are amongst the first features at presentation of these patients, and can occur together or separately (3, 14, 19, 32, 33).

We have recently investigated the clinical manifestations of a large international cohort of 191 patients with STAT3 GOF, including updated information on the originally described patients (4). While organ-specific autoimmunity can vary among patients, lymphoproliferation, described as hepatomegaly and/or splenomegaly and chronic reactive lymphadenopathy, is the most common clinical symptom, suggestive of a common mechanism underlying disease development (4, 5, 14, 34). Biopsies typically show lymphoid reactive hyperplasia. Malignancy is uncommon, but has been described in a small number of patients including marginal zone B cell lymphoma, large granular lymphocytic leukemia, and non-Hodgkin's lymphoma, suggesting an increased risk in this disease given the relatively small number of patients (3–5).

The severe lymphoproliferation observed in patients with STAT3 GOF has been described as a mimic of Autoimmune Lymphoproliferative Syndrome (ALPS). Within the large international cohort, three-quarters had lymphoproliferation manifested as diffuse lymphadenopathy (76%) and/or splenomegaly (72%). As with ALPS, elevated double negative (CD3 + CD4-CD8-) T cell populations appear to be the most reliable immune phenotype described in association with STAT3 GOF lymphoproliferation. Eighty-nine patients with lymphoproliferation within the large international cohort had DNTs assessed, and of those 82% had increased DNTs (4), which is not associated with Fas apoptosis defects (4, 5). The observation of elevated DNTs as an association with lymphoproliferative disease supports the quantification of DNTs to serve as a biomarker of STAT3 GOF activity. Autoimmune cytopenias are the second most common symptom and typically 2 to 3 cell lineages are affected. Direct anti-globulin (Coombs), anti-platelet or anti-granulocyte antibodies are present in 41% tested (4). Autoimmune cytopenias can be life-threatening requiring chronic transfusions, and are often refractory to standard treatment with IVIG and corticosteroids (14). Rituximab with steroid sparing immunosuppression, such as mycophenolate mofetil or sirolimus, can provide partial improvement but most patients with autoimmune cytopenias, fail to respond to these additional therapies(4). Janus kinase inhibitors (jakinibs) are often used as salvage therapy but have successfully controlled cytopenias (4, 34).

Post-natal growth failure, with usual profound short stature, was a hallmark symptom in the first descriptions of STAT3 GOF and one hypothesis is that this is secondary to defective STAT5b signaling (3, 5, 7, 14, 34). In the large international cohort, growth failure occurred in 57% of subjects (4). Over half of patients with growth failure (55%) had concurrent enteropathy or endocrinopathies, likely complicating their growth failure.

Interstitial lung disease is the most common non-infectious pulmonary manifestation in STAT3 GOF syndrome, and has been characterized as lymphocytic interstitial pneumonia, bronchiectasis, granulomatous lymphocytic interstitial lung disease (ILD) or cryptogenic organizing pneumonia (4, 14, 34, 35). Pulmonary disease can progress to pulmonary fibrosis, restrictive lung disease, and oxygen dependency. Overall survival is negatively affected when pulmonary disease has progressed to the point of oxygen dependency (4). ILD was also complicated by the immunodeficiency and accompanied by bronchiectasis in approximately one-quarter of patients with ILD. Patients with hypogammaglobulinemia and B cell lymphopenia were more likely to have bronchiectasis (4).

STAT3 GOF was initially discovered through genetic sequencing of a cohort of patients with early-onset type I diabetes, and this was initially considered a major feature of the disease (3). With identification of additional cases, it is evident that other endocrinopathies are more common feature such as hypothyroidism and growth hormone deficiency (3, 4, 7, 14, 33). Gastrointestinal manifestations primarily consist of enteropathy and/or colitis (4, 14, 19) presenting as diarrhea, bloody diarrhea, abdominal pain, achalasia, pseudo-obstruction, weight loss, and growth failure. Colonic biopsies show villous atrophy, small bowel thickening, and lymphocytic infiltration. In some cases, patients have been diagnosed with celiac disease, and gluten restriction has been helpful. In severe cases, patients have required total parenteral nutrition, which negatively affects survival (4). Autoimmune hepatitis has also been described in small number of patients, with liver transplant having mixed success (4, 34). Pancreatic exocrine insufficiency has also been reported in a small number of patients.

Inflammatory arthritis was also a major manifestation in the first descriptions of STAT3 GOF (5, 36). Oligoarthritis and polyarthritis mimicking juvenile idiopathic arthritis refractory to standard treatment have been reported. Eczema, psoriasis, and alopecia are frequently reported skin symptoms. Renal tubular disease and nephrolithiasis have been reported; chronic renal failure can be a cause of death (14, 37). Ophthalmologic disease has included ocular myasthenia gravis, uveitis, and papilledema (14, 38).

Vasculopathy and central nervous system (CNS) manifestations occur in a minority of patients. Systemic vasculitis, CNS vasculitis and stroke, pulmonary vasculitis, vascular malformations, and MoyaMoya Syndrome were recently reported (4). Other neurologic manifestations included isolated CNS lesions which presented as seizures and focal deficits. One patient responded to jakinib therapy (after failing corticosteroids) with improvement of symptoms despite no change on imaging (4).

Other clinically relevant immunologic abnormalities are varied in STAT3 GOF, but include humoral and cellular defects. Variable decreases in T, B, and NK cell quantities occur as do reduced functional responses. Lymphopenia and reduced functional lymphocyte responses more commonly occurred in patients receiving pulse or chronic steroids (4). Approximately half of patients have a humoral deficiency including hypogammaglobulinemia, B cell lymphopenia and reduced memory B cells (4, 5, 39, 40). A major caveat to immunophenotyping of the peripheral blood of patients is that this may not represent the local immune response at sites of inflammation/autoimmunity.

Infections are a common feature in STAT3 GOF, but infection susceptibility is complicated by the need for immune suppression to control autoimmune symptoms (4, 5, 10, 14, 34). Bacterial infections are most common followed by viral, fungal, opportunistic, and mycobacterial. Patients with hypogammaglobulinemia were at higher risk of bacterial infections while those with T cell lymphopenia were at higher risk of viral and/or fungal infections (4).

### Genotype-Phenotype correlation

Given the heterogeneity of STAT3 GOF syndrome symptoms, investigations attempting to predict the phenotype with specific disease manifestations based on genotype have been performed. In addition, Jagle et al. suggested a grouping based on luciferase activity. Although an important attempt to show characteristics for GOF based on transcriptional activity measured in the lab, not all variants could be categorized. Despite these efforts, a specific genotype-phenotype correlation has not been made (4, 6, 41). However, some gene domain-phenotype correlations were observed (4), as patients with SH2, CCD, and DBD variants were inclined to have endocrinopathies. Lymphoproliferative disease was a common feature across variants in all domains but more prominent in CCD (91%) and N-terminal domain variants (100%) (4). Although not reaching significance, Leiding et al. showed N-terminal domain conferred best survival and SH2 domain worst survival outcomes.

### Survival

Survival of patients with STAT3 GOF is impacted by many variables, but overall survival was recently described to be 88% (4). Age and number of organ systems affected did not impact overall survival, but time from first presentation to genetic diagnosis did. When time from presentation to diagnosis was shorter, overall survival was worse, suggesting those with milder phenotypes presumably taking longer to diagnose do better (4). The number of treatments a patient receives also impacts survival. Patients receiving 5 or more treatments fared worse, and showed that increasing number of treatments was a better indication of disease burden that the number of organ systems affected.

Specific disease manifestations also negatively affect survival. Progressive respiratory disease leading to oxygen dependency was highly associated with death. Enteropathy, total parenteral nutrition dependency, autoimmune hepatitis, and growth failure are also significantly associated with worse survival.

### Treatment approaches for STAT3 GOF

For many patients with JAK/STAT-GOF disorders and immune dysregulation, immunosuppression is the mainstay of therapy. Most patients require more than one conventional immunosuppressant such as corticosteroids in addition to steroid sparing agents such as rituximab, mycophenolate mofetil (MMF), sirolimus, tacrolimus, and alemtuzumab, which may provide partial responses. Other treatments include hormone replacement, anti-microbial prophylaxis, immunoglobulin replacement and hematopoietic stem cell transplant. In the international cohort of 191 patients, 77% received at least 1 immune suppressing medication and nearly half required 5 or more therapies (4). Despite these additional agents, most patients remained with uncontrolled disease. More than half of patients with cytopenias failed to respond to treatment with corticosteroids and immunoglobulin replacement. About a quarter of those who failed had been treated with rituximab in combination with MMF or sirolimus. Of those, approximately 80% had partial response and half required additional therapies.

Laboratory investigations have demonstrated that STAT3 GOF leads to increased transcriptional activity following activation. This mechanistic insight provides rationale for blocking upstream signals that lead to STAT3 phosphorylation. Because of the limited therapeutic options for these patients and the fact that they often fail conventional immunosuppressants, we and others began to use jakinibs as targeted therapy. In 2018, Forbes et al. reported 6 STAT3 GOF patients who had clinical improvement with ruxolitinib (5 patients) and tofacitinib (1 patient). Most patients experienced a reduction in autoimmune disease activity, to include enteropathy, as well as the reversal of severe, life-threatening interstitial lung disease and autoimmune cytopenias (34, 42). IL-6 blockade with tocilizumab, an IL-6R antagonist, has shown to alleviate arthritis and autoimmune complications in a small subset of patients (5, 26, 34). In our large international cohort, 19% of patients received jakinibs as salvage therapy. Use of jakinibs, sometimes in combination with tocilizumab led to control of cytopenias and oxygen withdrawal in patients with interstitial lung disease. Jakinibs also improved optic neuritis in 1 patient, clinical improvement in 1 patient with a cerebral vascular lesion, and control of enteropathy with withdrawal of total parenteral nutrition. While precision therapy with jakinibs and IL-6 blockade are reasonable based on mechanism and published experiences, clinical trials are needed to assess disease-specific safety and efficacy. That being said, jakinibs offer a viable option with published reports supporting their use when life-threatening manifestations are not controlled with standard immune suppression in patients with STAT3 GOF syndrome.

Hematopoietic stem cell transplant (HSCT) has been used as life-saving therapy for treatment refractory disease manifestations (manuscript in progress). HSCT is considered in patients with progressive disease where medication management has not achieved optimal control of disease. Survival is approximately 62% (4). An effort to report the international experience in HSCT in STAT3 GOF syndrome is underway to understand what influences transplant survival and how disease manifestations at the time of transplant interfere with outcomes.

## Summary

Investigation of monogenic causes of errors of the immune response has provided tremendous insight into human immunity, enabling novel diagnoses for patients and providing hope for precision therapies based on the molecular mechanisms of disease. Since initially described in 2014, identification of additional patients with STAT3 GOF syndrome and studies of the mechanisms behind this disease have enhanced our understanding of the immune dysregulation found in these patients. Early identification of disease is essential given the heterogeneity of disease manifestations with substantial morbidity. This disorder should be suspected in patients with early onset and progressive autoimmunity. Lymphoproliferation is the most prominent feature with most patients presenting early in life with splenomegaly and autoimmune cytopenias. Precision therapy offers an optimal treatment in order to control disease manifestations. However, there are still many remaining questions regarding the molecular mechanisms of increased STAT3 transcriptional activity in the spectrum of disease-causing variants across different protein domains, in dissecting infectious susceptibility due to STAT3 GOF from that due to immunomodulatory treatments, and in the design of clinical trials to address unmet therapeutic needs for these patients.
